# Nurse-Filled versus Pharmacy-Filled Medication Organization Devices—Survey on Current Practices and Views of Home Care Nursing Services

**DOI:** 10.3390/healthcare10040620

**Published:** 2022-03-25

**Authors:** Thomas Schmid, Falk Hoffmann, Michael Dörks, Kathrin Jobski

**Affiliations:** 1 Faculty of Social and Health Studies, University of Applied Sciences Kempten, 87435 Kempten, Germany; thomas.schmid@hs-kempten.de; 2 Department of Health Services Research, Faculty VI Medicine and Health Sciences, Carl von Ossietzky University Oldenburg, 26129 Oldenburg, Germany; falk.hoffmann@uni-oldenburg.de (F.H.); michael.doerks@uni-oldenburg.de (M.D.)

**Keywords:** medication organization device, medication adherence, mediation errors, inter-professional practice, home care, nursing services, long-term care, medication management, blister pouches, blister cards

## Abstract

Medication organization devices (MODs) are widely used among home care nursing services. However, current practices such as the responsibility for filling MODs, different MOD types used and requirements of home care nursing services are largely unknown. The study aimed at analyzing home care nursing services’ current practices regarding MOD use, investigating their requirements and determining whether different practices met these requirements. A survey was administered online to German home care nursing services in February 2021. The importance of requirements and the extent of satisfaction were measured using a five-point scale. Attitudes towards disposable, pharmacy-filled MODs were recorded as free text. In total, 690 nursing services responded (67.5% privately owned and 34.5% based in large cities), 92.2% filled MODs themselves and used predominantly reusable, rigid MODs. Pharmacies filling MODs used primarily disposable MODs. Satisfaction with current practices was generally high. Respondents filling MODs themselves were more satisfied with nurses’ medication knowledge, but less satisfied with cost effectiveness than those who had pharmacies fill MODs. Of all respondents filling MODs themselves who expressed an opinion on disposable, pharmacy-filled MODs, 50.9% were skeptical, primarily due to fear of losing flexibility. However, no difference in satisfaction with flexibility was found between respondents filling MODs themselves and those using pharmacy-filled MODs. In conclusion, employment of MODs in the professional care setting is a complex task with nursing services as key constituents. There is potential for improvement in the inter-professional collaboration between pharmacies and home care nursing services on the use of MODs. Measures for improvement have to address home care nursing services’ requirements with respect to flexibility and medication knowledge.

## 1. Introduction

Especially in the US, Western Europe and Japan, the population is ageing rapidly [1], turning multimorbidity and consequently polypharmacy into a global health challenge [2]. Not surprisingly, polypharmacy is particularly prevalent among recipients of professional care [3,4].

To support patient adherence to complex drug regimens, medication organization devices (MODs) are widely used, and provisioning is reimbursed under certain conditions [5,6,7,8,9,10]. In Germany, where this study has been carried out, 99.3% of nursing homes reported in 2017 to use at least one of the five most common types of MODs [11]. Often also referred to as ”medication organizers“, “monitored dosage systems”, “dose administration systems” and “drug reminder packaging” [12,13,14,15], these devices are prepared by removing oral solid dosage forms from original packs and repackaging them into partitions of a new container, with each compartment reflecting a dose-taking time [12]. The most common systems are reusable, multi-compartment, cassette-format MODs made from rigid, thermosetting polymers and disposable MODs made from thermoplastics, thus easier to seal. The latter come in the formats of blister pouches (also known as sachets or strip packaging), blister cards with a cardboard frame (also known as punch cards or “bingo” cards) and foil-sealed cups [16,17,18]. Some of the disposable MODs are marketed as capable of accommodating liquids [13].

Dispensing medication into MODs is an important and responsible task, given that it is a potential source of medication errors, and its organization may have an impact on error frequency [10,19,20,21,22]. In the professional care setting, filling MODs may either be performed by nursing staff or pharmacies, with disposable MODs usually being provisioned by pharmacies [16], which may be attributed to substantial capital investments required for automating the filling process and sealing. In Germany, where this study was conducted, many pharmacies choose to commission the filling of disposable MODs to dedicated repackaging facilities.

While considerable research has been undertaken on the effectiveness of individual MOD types and the collaboration between nurses and pharmacists to improve adherence [23,24,25,26,27,28,29,30,31,32,33,34], little is known about the current practices of MOD provisioning from the perspective of professional caregivers. This applies to the prevalence of the various MOD types, the number of pharmacies involved, quality assurance measures, the needs of professional caregivers related to providing long-term medication as well as the suitability of pharmacy-filled MODs to meet the professional caregivers’ needs. What is more, medication organization devices have been associated with a removal of knowledge from caregivers [7], but so far it has been unclear whether this varies with different practices of provisioning medication organizers. This study aims at closing these research gaps for German home care nursing services.

## 2. Materials and Methods

### 2.1. Design and Recruitment

We conducted a nationwide cross-sectional survey of German home care nursing services. In January 2021, 14,378 home care nursing services were identified via the nursing service registry of Germany’s largest statutory health insurance AOK. Overall, 8991 entries included email addresses to which an invitation and a link to an online version of the survey was sent in February 2021. The invitation addressed both the services’ management and the services’ head of nursing as eligible to participate. No exclusion criteria were defined. The survey was delivered to 6913 addressees; failure to deliver the remaining messages was largely attributable to rejection of specified external emails, full mailboxes and invalid email addresses. Two weeks after the initial message, the recipients received an email reminder. The survey period ended another two weeks later.

### 2.2. Survey Development and Content

A 21-item survey was developed based on qualitative interviews with home care nursing services from a pre-study, existing MOD literature and a recent German study on practices and needs of German nursing homes related to MODs [11]. The survey language was German; a translation of the complete survey can be found in the supplement. All questions but one were closed-ended. Where these questions referred to attitudes or judgements, the order of response options was randomized.

Survey items included nursing service characteristics such as ownership, community size and location (federal state) as well as the number of care recipients and the percentage of clients with a doctor’s prescription for MOD provisioning. Further items addressed whether nursing services dispensed medication into MODs themselves or had pharmacies fill MODs and which MOD type was used.

For home care nursing services filling MODs themselves, additional questions included the number of different drugs dispensed per client, the filling time and quality checks. The open-ended question asked the home care nursing services in this group to justify their attitudes towards disposable, pharmacy-filled MODs in their own words.

The importance of quality, organizational, economic and legal requirements of home care services with respect to providing long-term medication was determined using a five-point scale, ranging from “not at all important” to “very important”. Likewise, encompassing options from “very poor” to “very good”, the extent of satisfaction with current practices was measured. In addition to these five-point scales, the survey provided the option “no opinion/don’t know” to skip a selection.

### 2.3. Statistical Analysis

Descriptive statistics (percentages, median and interquartile range (IQR)) were used to present characteristics of home care nursing services stratified by filling responsibility (nurse-filled vs. pharmacy-filled).

Furthermore, the subset of nursing services filling medication into MODs themselves was analyzed with respect to their practices and their attitudes towards disposable, pharmacy-filled MODs. The responses to the open-ended question on these attitudes were examined by constructing a system of categories.

The categorical frequencies (five-point scaled ratings) referring to the importance of the pre-determined criteria and the extent of satisfaction with current practices were compared between nurse-filled and pharmacy-filled MODs, using Fisher’s exact test for all comparisons. Only valid answers were included, thus e.g., nursing services answering “no opinion/don’t know” were excluded from the respective comparisons.

All analyses were performed using SAS for Windows Version 9.4 (SAS Institute Inc., Cary, NC, USA).

## 3. Results

### 3.1. Respondent Characteristics

Overall, 690 home care nursing services responded to the survey. Of those, 67.5% were privately owned, 28.1% were owned by a non-profit organization and 4.3% in public ownership (Table 1). Of all nursing services, 34.5% reported to be based in cities with a population exceeding 100,000, while 7.5% were located in communities with a population of less than 5000 inhabitants. The majority of nursing services was based in Bavaria (Supplementary Figure S1). The median number of clients amounted to 105 (IQR: 70–175).

### 3.2. Prevalence of MODs, MOD Types and Filling Responsibilities

The median percentage of clients receiving a prescription for an MOD was 40% (IQR: 20–60%).

For clients receiving a prescription for the provisioning of an MOD, respondents reported to use predominantly reusable, rigid MODs; 91.9% of the respondents primarily worked with such MODs. Only 3.0% of the respondents used primarily blister pouches, 2.3% blister cards, 1.9% unsealed dosage cups and 0.6% foil-sealed cups.

With 636 (92.2%), dispensing medication into MODs was largely performed by nursing services themselves, while 54 (7.8%) had MODs filled by pharmacies. When pharmacies filled MODs, they used markedly different MOD types. While 97.8% of nursing services who filled MODs themselves used reusable rigid MODs, this was the case in only 22.2% of those who had pharmacies fill the MODs. In contrast, 75.9% of the nursing services using pharmacy-filled MODs, received blister pouches, blister cards or foil-sealed cups, hence sealed, disposable MODs.

### 3.3. Current Practices and Attitudes of Nursing Services Filling MODs

As displayed in Table 2, the median number of pharmacies supplying original packs to nursing services was 3 (IQR: 1–5). Overall, 355 (55.8%) of these nursing services stated that filling MODs took place at the care recipient’s residence. Of the remaining 281 respondents who filled MODs at the nursing service office, 48.0% had the same nurse who dispensed the drugs into MODs check them for conformity with the medication list, 31.0% had another nurse check the MODs at the nursing service office and 21.0% at the care recipient’s residence. The median number of different drugs per care recipient filled into MOD’s amounted to 6 (IQR: 5–8) and the median filling time per care recipient and week was 15 (IQR: 10–20) minutes.

In total, 597 nursing services filling MODs expressed an opinion regarding disposable, pharmacy-filled MODs. Of those, 25.5% did not know how switching to disposable, pharmacy-filled MODs would change reimbursement, 21.3% believed they could keep on invoicing payers, but would have to pay pharmacies a fee, the remaining 53.3% believed they would have to stop invoicing payers. About half of the 597 nursing services were skeptical towards disposable, pharmacy-filled MODs, whereas 14.6% had a positive and 34.5% a neutral opinion, respectively.

Between nursing services with different attitudes, no substantial differences were observed with respect to most characteristics (Supplementary Table S1). However, nursing services with a skeptical attitude more often had MODs checked for conformity by the same nurse (51.5%) than those with a neutral or positive attitude (40.9% and 43.9%, respectively). Moreover, nursing services with a skeptical attitude less often believed that they could keep on invoicing when transitioning to disposable, pharmacy-filled MODs (15.5%) than those with a neutral or positive attitude (20.9% and 42.5%).

Of the 597 nursing services filling MODs and expressing an opinion on disposable, pharmacy-filled MODs, 583 answered the open-ended question to justify their attitudes towards disposable, pharmacy-filled MODs (Supplementary Table S2). The by far most common category turned out to be the fear of a loss of flexibility when switching to disposable, pharmacy-filled MODs (224 mentions).

### 3.4. Nursing Services’ Requirements for Providing Long-Term Medication

All eight pre-determined criteria were considered important or very important by a vast majority of respondents (Supplementary Table S3). Quality of care/error avoidance were rated this way by 97.9%, and flexibility in case of medication changes by 95.8% of respondents. Overall, 90.3% deemed nurses’ medication knowledge important or very important, and 87.7% did so for cost effectiveness.

**Figure 1:**
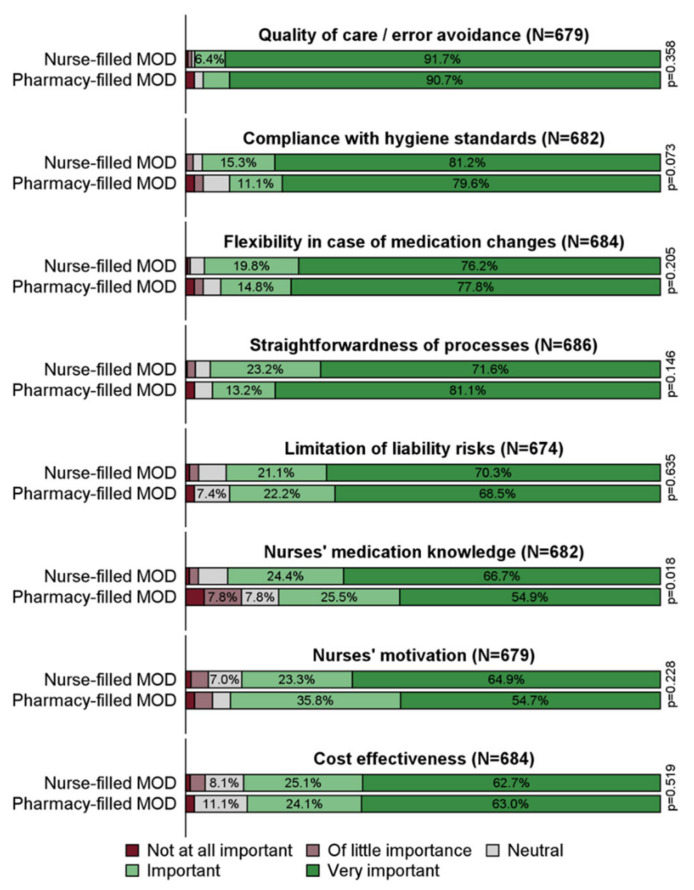
How important are the respective criteria when providing long-term medication? Answers by filling responsibility, N = number of valid answers for the respective item. MOD: medication organization device.

As shown in Figure 1, no statistically significant difference between the ratings of nurse-filled and pharmacy-filled MODs was found except for nurses’ medication knowledge (*p* = 0.018). Respondents who filled MODs themselves were more likely to consider this criterion important or very important than those using pharmacy-filled MODs (91.1% vs. 80.4%).

### 3.5. Satisfaction with Current Practices

Satisfaction with the extent to which current practices met requirements was in general high. Overall, 92.8% reported that compliance with hygiene standards was good or very good (Supplementary Table S3). Nurses’ medication knowledge was good or very good for 74.1% of respondents. The lowest levels of satisfaction were recorded for flexibility in case of medication changes with 68.9% and cost effectiveness with 51.2% of respondents, respectively.

**Figure 2:**
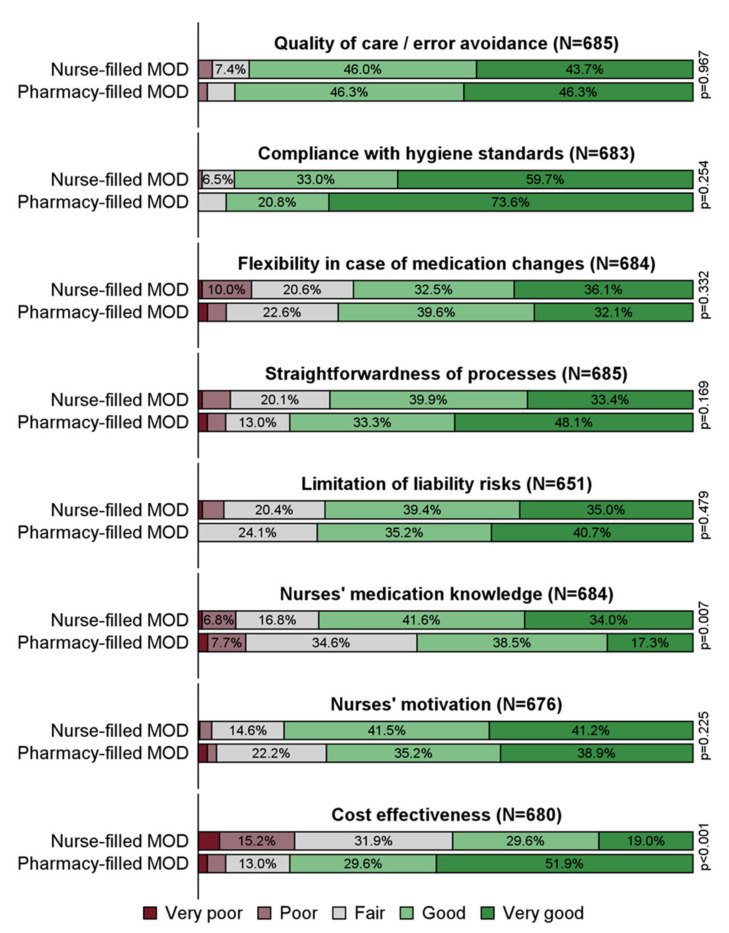
How well do your current practices fulfill the respective criteria for providing long-term medication? Answers by filling responsibility, N = number of valid answers for the respective criterion. MOD: medication organization device.

As displayed in Figure 2, statistically significant differences between the satisfaction with nurse-filled and pharmacy-filled MODs could be observed for only two criteria. Nursing services who filled MODs themselves were more likely to be satisfied with their nurses’ medication knowledge than those having pharmacies fill the MODs (*p* = 0.007). Conversely, nursing services using pharmacy-filled MODs were more likely to be satisfied with cost effectiveness (*p* < 0.001).

Considering only the 54 nursing services using pharmacy-filled MODs, there was some satisfaction variability within MOD types (Supplementary Figure S2). Among the disposable MODs, nursing services tended to be more satisfied with the flexibility of blister cards than with that of pouches and foil-sealed cups. Even more variability was found for satisfaction with nurses’ medication knowledge. While 86.7% of nursing services who received blister cards reported good or very good satisfaction, only 40.0% and 0% did so for blister pouches and foil-sealed cups.

## 4. Discussion

### 4.1. Key Findings

This study shows the current practices regarding the MOD use in German home care nursing services. The vast majority of nursing services dispensed long-term medication for care recipients with a prescription for this service into MODs themselves instead of having a pharmacy fill MODs. When comparing the two groups’ ratings with respect to the importance of pre-defined requirements, no differences were observed except that nursing services filling MODs themselves were more likely to find nurses’ medication knowledge important or very important. Moreover, satisfaction with nurses’ medication knowledge was significantly higher in nursing services who filled MODs themselves, whereas nursing services using pharmacy-filled MODs were more likely to be satisfied with cost effectiveness.

### 4.2. Interpretation

Prevalence of MOD types and low share of pharmacy-filled MODs.

This study found that nursing services who filled MODs themselves used predominantly reusable, rigid MODs, while those who had pharmacies fill MODs received primarily disposable MODs. As previous research already suggested this pattern and linked it to higher feasible levels of automation for disposable MODs [16], this result was not surprising. One of the striking results of this study, however, is the low share of home care nursing services who chose to have MODs filled by pharmacies, especially when considering that respondents evaluated this approach as more cost effective than dispensing medication into MODs themselves. This result poses a strong contrast to recent findings for German nursing homes, where 44% of the facilities had MODs filled by pharmacies [11].

One could assume more complex drug regimens in nursing homes residents an obvious explanation, however, the number of long-term medications in a cross-sectional study of nursing homes in Northwestern Germany (mean of 6.3) did not differ from our findings [35]. Other reasons might comprise differences in the organizational requirements of nursing homes and home care nursing services, differences in the availability of skilled employees, as well as reimbursement and legal issues. While for instance in the nursing home setting, pharmacies filling MODs can provide patient information leaflets to the ward(s), in the decentral home care setting an extra effort is required to make sure that the leaflets in their most current versions are available at the patients’ residences. Further, this study has brought to light a considerable insecurity with respect to reimbursement when transitioning to pharmacy-filled MODs. Finally, one might argue that the lack of a clear legal framework is the key reason why pharmacy-filled MODs have so far only occupied a niche position in the German home care setting. This view builds on the fact that, to date, according to the German Pharmacy Act pharmacies are legally required to close contracts clearly specifying rights and duties when dealing with nursing homes, but not so for home care nursing services.

### 4.3. Flexibility in Case of Medication Changes

Of all respondents, 95.8% considered flexibility important or very important, but only 68.9% rated their current practices as good or very good, making flexibility the requirement with the second largest discrepancy between importance and satisfaction ratings. In addition, satisfaction with flexibility in this study was substantially lower than in the German nursing home setting (87.1%) [11]. When differentiating between nurse-filled and pharmacy-filled MODs, the results of this study on flexibility also play into the observed share of pharmacy-filled MODs. Among home care services filling MODs themselves a potential lack of flexibility in case of medication changes was the most common argument against transitioning to disposable, pharmacy-filled MODs. This finding is in line with concerns of the “dispensary becoming remote from the customers”, as expressed in a study determining prevalence and potential harm of prescribing, monitoring, dispensing and administration errors in UK care homes [19]. However, our study showed no difference in satisfaction with flexibility between nurse-filled and pharmacy-filled MODs. Based on the conjunction of these findings, the authors infer that flexibility related reservations of nursing services who currently fill MODs themselves against pharmacy-filled MODs are not justified. How MCA-filling pharmacies managed to meet the flexibility needs of home care nursing services to the same extent as nursing services who filled MCAs themselves has not been investigated in this research. Measures might include short delivery cycles or, in case an intervention by the nursing service or the treating physician is required to implement a medication change, the use of repair kits to re-seal previously opened disposable MCAs.

### 4.4. Nurses’ Medication Knowledge

Previous research has found removal of knowledge from caregivers a key issue to consider when working with MODs [7], but so far little is known about which MOD-related practices reinforce medication knowledge among professional caregivers. This study has found a significantly higher satisfaction of nursing services with their staff’s medication knowledge for those services who dispensed medication into MODs themselves than for those who had pharmacies dispense medication into MODs. This is likely to be attributable to nursing services believing that it helps their staff to keep their patients’ medication lists and appearance of drugs in mind when filling MODs themselves. Considering the usage patterns observed in other research [36], mobile electronic medical records should be well suited to provide medication lists and appearance of drugs. Yet, to implement this, further improvements to these systems e.g., in terms of user-friendliness seem essential [37,38,39].

The results of our study on both key issues, flexibility and nurses’ medication knowledge, are coherent with other research that has found a lack of understanding of each other’s needs, roles and capabilities among nurses and pharmacists in general [34,40,41], and in the specific context of MODs [21]. Therefore, a regular exchange between both professions, e.g., by means of interdisciplinary training, should be taken into consideration. As a case in point, a Norwegian study investigated nurses’ and pharmacists’ perceived learning experience after participating in inter-professional medication reviews (IMRs) in primary health care for up to two years [31]. Participants reported that IMRs contributed to improving their own practice and the quality of drug management.

### 4.5. Strengths and Limitations

As a major strength, this study is the first to provide comprehensive information about the current practices of MOD provisioning, the importance of requirements when providing long-term medication and the extent of satisfaction with current practices from the perspective of German home care nursing services. Limitations include that several items such as the number of drugs per recipient or the time needed to fill MODs were based on the participants’ estimates and could not be verified. Further, this study was not designed to delve into the underlying reasons for the status quo or for nursing services’ satisfaction ratings of different filling approaches. The major limitation, however, is the low response, which in part was due to failures in mail delivery processes including invalid addresses. Moreover, and probably attributable to the geographical proximity to the university conducting the survey, a disproportionally high number of respondents were located in the state of Bavaria, limiting the generalizability of the results.

## 5. Conclusions

The employment of MODs in the professional care setting is a complex task, with nursing services as key constituents exhibiting numerous requirements that different practices and MOD types meet to different degrees. In our study, the vast majority of home care nursing services dispensed long-term medication themselves into MODs rather than having a pharmacy fill MODs. Concerns of losing flexibility and nurses’ medication knowledge have been identified as potential obstacles in transitioning to pharmacy-filled MODs. With respect to these concerns, this study has revealed a potential and measures for improvement in the inter-professional relationship between pharmacies and home care nursing services. Finally, future studies should examine the underlying reasons for the status quo and gain further insight into dispensing practices in home care nursing services.

## Figures and Tables

**Table 1:** Characteristics of home care nursing services providing long-term medication for care recipients with an MOD prescription, by filling responsibility.

	MODs are Filled by		Overall
	Nursing Service (*N* = 636)	Pharmacy (*N* = 54)	(*N* = 690)
Type of ownership			
Private	435 (68.4%)	31 (57.4%)	466 (67.5%)
Non-profit	174 (27.4%)	20 (37.0%)	194 (28.1%)
Public	27 (4.2%)	3 (5.6%)	30 (4.3%)
Size of the city, where the nursing service is located (population)			
>100,000	220 (34.6%)	18 (33.3%)	238 (34.5%)
20,000 -≤ 100,000	208 (32.7%)	25 (46.3%)	233 (33.8%)
5000 -< 20,000	160 (25.2%)	7 (13.0%)	167 (24.2%)
<5000	48 (7.5%)	4 (7.4%)	52 (7.5%)
Median number of care recipients (IQR)	103.5 (70–170)	110 (72–220)	105 (70–175)
Median percentage of care recipients with a prescription for an MOD (IQR)	40% (20–60%)	35% (16–65%)	40% (20–60%)
MOD types *			
Reusable rigid MOD	622 (97.8%)	12 (22.2%)	634 (91.9%)
Unsealed dosage cup	12 (1.9%)	1 (1.9%)	13 (1.9%)
Blister pouch	-	21 (38.9%)	21 (3.0%)
Blister card	-	16 (29.6%)	16 (2.3%)
Foil-sealed cup	-	4 (7.4%)	4 (0.6%)

* 1 nursing service reported use of “blisters”, 1 nursing service reported to use only syringes for administration via tube (intensive nursing care). IQR: interquartile range; MOD: medication organization device.

**Table 2:** Characteristics of home care nursing services filling MODs.

	Overall (*N* = 636)
Median number of pharmacies involved in the medication supply (IQR)	3 (1–5)
Site where MODs are filled	
Residence of the care recipient	355 (55.8%)
At the nursing service office	281 (44.2%)
The correct filling of MODs is usually checked by	
The same nurse	135 (48.0%)
Another nurse (at the nursing service office)	87 (31.0%)
Another nurse (at the residence of the care recipient)	59 (21.0%)
Median time needed to fill MODs per care recipient and week in minutes (IQR) *	15 (10–20)
Median number of different drugs per care recipient (IQR)	6 (5–8)
Nursing services expressing an opinion regarding disposable, pharmacy-filled MODs **	(*N* = 597)
Expected changes in reimbursement if MODs were filled by a pharmacy	
Unknown	152 (25.5%)
Filling could be invoiced by the nursing service, pharmacy would receive a fee	127 (21.3%)
Filling could no longer be invoiced by the nursing service	318 (53.3%)
Attitude towards disposable, pharmacy-filled MODs	
Positive	87 (14.6%)
Neutral	206 (34.5%)
Skeptical	304 (50.9%)

IQR: interquartile range; MOD: medication organization device. * excluding picking up prescriptions and drugs as well as delivery to the client when MODs were filled at the office. ** 39 nursing services did not express an opinion regarding disposable, pharmacy-filled MODs.

## Data Availability

The data that support the findings of this study are available from the corresponding author on reasonable request.

## Supplementary Materials

The following supporting information can be downloaded at: https://www.mdpi.com/article/10.3390/healthcare10040620/s1, Figure S1: Location (federal state) and type of ownership of home care nursing services by filling responsibility, Figure S2: How well do pharmacy-filled MODs fulfill “Nurses’ medication knowledge” and “Flexibility in case of medication changes”, Answers by MOD types; Table S1: Characteristics of nursing services filling MODs themselves by their attitudes towards disposable, pharmacy-filled MODs, Table S2: Categorized arguments in favor of or against disposable pharmacy-filled MODs in nursing services filling MODs themselves, Table S3: How important are the respective criteria and how well do your current practices fulfill the criteria when providing long-term medication, by filling responsibility, survey instrument.
