# Supplementary material for: Nurse-Filled versus Pharmacy-Filled Medication Organization Devices—Survey on Current Practices and Views of Home Care Nursing Services
Source: Healthcare (Basel). 2022 Mar 25;10(4):620. doi: 10.3390/healthcare10040620 (PMC9028845; doi:10.3390/healthcare10040620)
Supplement: Supplementary file 1 [file healthcare-10-00620-s001.zip › healthcare-1636422-supplementary.pdf]

Supplementary Material

Supplementary Table S1: Characteristics of nursing services filling MODs themselves by their attitudes towards disposable, pharmacy-filled MODs.

	**Positive (N=87)**	**Neutral (N=206)**	**Skeptical (N=304)**
Type of ownership			
Private	59 (67.8%)	134 (65.1%)	209 (68.8%)
Non-profit	27 (31.0%)	64 (31.1%)	77 (25.3%)
Public	1 (1.2%)	8 (3.9%)	18 (5.9%)
Size of the city (inhabitants)			
> 100,000	27 (31.0%)	80 (38.8%)	101 (33.2%)
20,000 -≤ 100,000	26 (29.9%)	62 (30.1%)	110 (36.2%)
5,000 -< 20,000	30 (34.5%)	49 (23.8%)	67 (22.0%)
< 5,000	4 (4.6%)	15 (7.3%)	26 (8.6%)
Median number of care recipients (IQR)	110 (70-200)	100 (60-170)	108 (75-176.5)
Median percentage of care recipients with a prescription for an MOD (IQR)	40% (22%-65%)	40% (20%-60%)	40% (20%-62.5%)
Median number of pharmacies involved in the medication supply (IQR)	3 (1-5)	3 (2-5)	2.5 (1-5)
Site where MODs are filled			
Residence of the care recipient	46 (52.9%)	118 (57.3%)	168 (55.3%)
At the nursing service office	41 (47.1%)	88 (42.7%)	136 (44.7%)
When filled at the nursing service office, the correct filling of MODs is usually checked by	(N=41)	(N=88)	(N=136)
The same nurse	18 (43.9%)	36 (40.9%)	70 (51.5%)
Another nurse (at the nursing service office)	14 (34.2%)	30 (34.1%)	41 (30.2%)
Another nurse (at the residence of the care recipient)	9 (22.0%)	22 (25.0%)	25 (18.4%)
Median time needed to fill MODs per care recipient and week in minutes (IQR)	15 (10-20)	15 (10-20)	15 (10-19)
Median number of different drugs per care recipient (IQR)	6 (5-8)	6 (5-8)	7 (5-8)
Expected changes in reimbursement if MODs were filled by a pharmacy			
Unknown	14 (16.1%)	59 (28.6%)	79 (26.0%)
Filling could be invoiced by the nursing service, pharmacy would receive a fee	37 (42.5%)	43 (20.9%)	47 (15.5%)
Filling could no longer be invoiced by the nursing service	36 (41.4%)	104 (50.5%)	178 (58.6%)

39 nursing services did not express an opinion regarding disposable, pharmacy-filled MODs
IQR: interquartile range; MOD: medication organization device

Supplementary Table S2: Categorized arguments in favor of or against disposable pharmacy-filled MODs in nursing services filling MODs themselves.

Arguments in favor of disposable pharmacy-filled MODs	N	Arguments against disposable pharmacy-filled MODs	N
Reduced risk of medication errors	48	Loss of flexibility, extra work in case of medication changes	224
Reduction of workload	33	Loss of medication knowledge, competencies	88
Time savings	30	Packaging waste, environmental concerns	46
Cost effectiveness	24	Liability issues	46
Hygiene	20	Conformity checks unclear, causing additional work	45
Straightforwardness	16	Loss of revenues / additional cost	25
Positive attitudes, experiences in the past	11	Reimbursement issues	22
Reduced liability risk, accountability	10	Increased risk of medication errors	21
Clarity	5	No relevant saving of time, effort	20
Good labelling and documentation	4	Negative attitudes, experiences in the past	19
Simplification of conformity checks and medication management	2	Lack of trust in / fear of dependency from pharmacies	18
		Requires more time, effort	18
		Communication challenges	16
		Difficult to handle for clients	16
		Lack of medication overview	15
		Loss of patient contact	14
		Offerings by pharmacies not sufficient	14
		Drugs not taken have to be disposed	13
		Not suitable for all dosage forms	5
		Less work for employees	3
		Declined by medical review boards of the statutory health insurances	3
		Pharmacies lack patient knowledge	1
		System too rigid	1
		Limited patient choices	1

Based on 583 answers. Answers could be categorized into different arguments.
MOD: medication organization device

Supplementary Table S3: How important are the respective criteria and how well do your current practices fulfill the criteria when providing long-term medication, by filling responsibility.

	Criterion is considered important or very important			Satisfaction with current practices rated good or very good		
	Nursing service (N=636)	Pharmacy (N=54)	Overall (N=690)	Nursing service (N=636)	Pharmacy (N=54)	Overall (N=690)
Quality of care / error avoidance	613 (98.1%)	52 (96.3%)	665 (97.9%)	566 (89.7%)	50 (92.6%)	616 (89.9%)
Compliance with hygiene standards	606 (96.5%)	49 (90.7%)	655 (96.0%)	584 (92.7%)	50 (94.3%)	634 (92.8%)
Flexibility in case of medication changes	605 (96.0%)	50 (92.6%)	655 (95.8%)	433 (68.6%)	38 (71.7%)	471 (68.9%)
Straightforwardness of processes	600 (94.8%)	50 (94.3%)	650 (94.8%)	463 (73.4%)	44 (81.5%)	507 (74.0%)
Limitation of liability risks	567 (91.5%)	49 (90.7%)	616 (91.4%)	444 (74.4%)	41 (75.9%)	485 (74.5%)
Nurses' medication knowledge	575 (91.1%)	41 (80.4%)	616 (90.3%)	478 (75.6%)	29 (55.8%)	507 (74.1%)
Nurses' motivation	552 (88.2%)	48 (90.6%)	600 (88.4%)	514 (82.6%)	40 (74.1%)	554 (82.0%)
Cost effectiveness	553 (87.8%)	47 (87.0%)	600 (87.7%)	304 (48.6%)	44 (81.5%)	348 (51.2%)

Percentages refer to the number of valid answers for the respective criterion

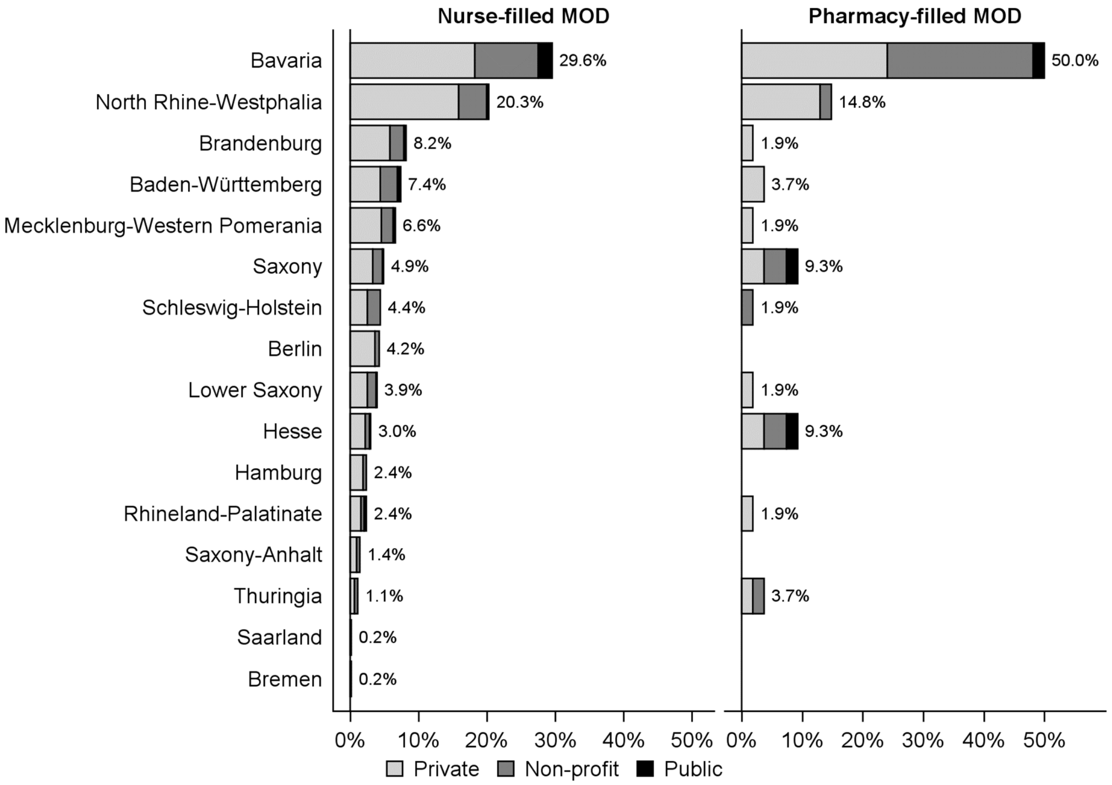

Supplementary Figure S1: Location (federal state) and type of ownership of home care nursing services by filling responsibility

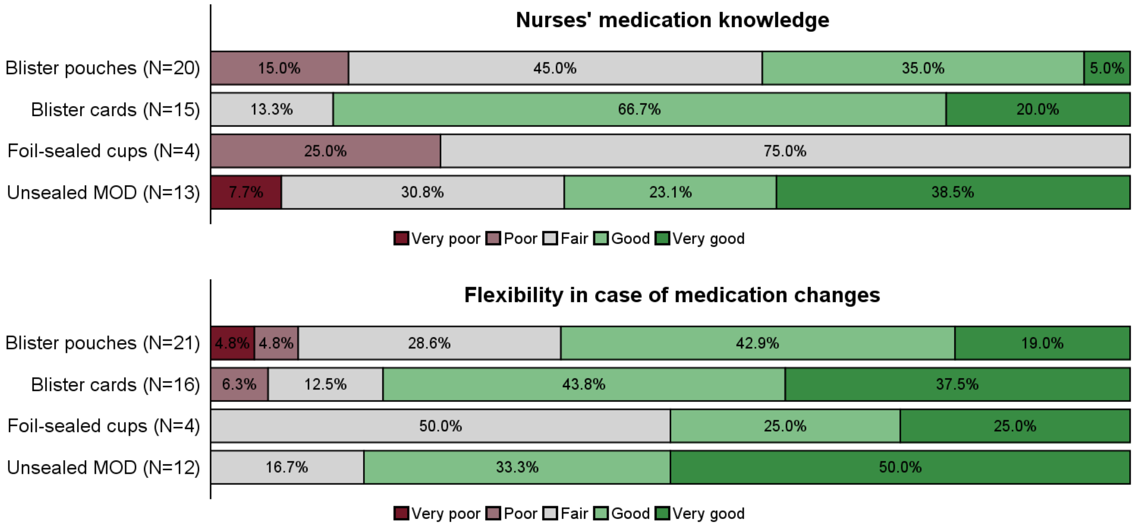

Supplementary Figure S2: How well do pharmacy-filled MODs fulfill "Nurses' medication knowledge" and "Flexibility in case of medication changes", Answers by MOD types

MOD: medication organization device
N = number of valid answers for the respective MOD among home care nursing services who had pharmacies fill MODs
Unsealed MODs include reusable rigid MOD and unsealed dosage cups.

Supplementary information - survey instrument

The following survey has been translated from German. *Blue, italicized items reflect survey structure and design features that were not visible to respondents.*

Survey on medication practices of home care nursing services

I. This initial section is intended to investigate what percentage of your clients receives a prescription for the filling of a medication organization device and, if a prescription has been issued, how filling the device is carried out. Please answer the following questions:

   1. What percentage of your clients receives a prescription for the filling of a medication organization device? *(answer validation: numbers between 0 and 100% were accepted)*

      All subsequent questions refer to the long-term medication of your clients, only, since the provision of "as required" drugs is often handled highly individually.

   2. When filling of a medication organization device has been prescribed for one of your care recipients: who usually fills the device?
      - ☐ employees of the home care nursing service
      - ☐ a pharmacy

      (answer validation: only one box could be ticked. If „employees of the home care nursing service" was ticked, the survey continued with section IV)

II.

   1. Into what type of medication organization device does the pharmacy usually fill your clients' long-term medication (please tick only one box)?
      - ☐ Reusable rigid medication organization device (commonly referred to as "pillbox")
      - ☐ Unsealed dosage cup
      - ☐ Blister pouch
      - ☐ Blister card
      - ☐ Foil-sealed cup

      (answer validation: only one box could be ticked)

III. This section is supposed to investigate the importance of various criteria for your home care nursing service when providing long-term medication to your clients and to research how well your current practices meet these criteria. Please answer the following questions:

1. How important are the following criteria for your homecare nursing service when providing long-term medication to your care recipients?
   - cost effectiveness
   - quality of care / error avoidance
   - flexibility in case of medication changes
   - limitation of liability risks
   - nurses‘ motivation
   - nurses‘ medication knowledge
   - straightforwardness of processes
   - compliance with hygiene standards

Answer options for each criterion entailed „not at all important", "of little importance", "neutral", "important", "very important" and "no opinion/don't know". *(sequence of criteria was randomized)*

2. How well do your current practices of providing long-term medication to your clients meet the criteria?
   - cost effectiveness
   - quality of care / error avoidance
   - flexibility in case of medication changes
   - limitation of liability risks
   - nurses‘ motivation
   - nurses‘ medication knowledge
   - straightforwardness of processes
   - compliance with hygiene standards

Answer options for each criterion entailed „very poor", "poor", "fair", "good", "very good" and "no opinion/don't know". *(sequence of criteria was randomized, survey continues with section X)*

IV.

1. Into what type of medication organization device do you usually fill your clients‘ long-term medication (please tick only one box)?
   - ☐ reusable rigid medication organization device (commonly referred to as "pillbox")
   - ☐ unsealed dosage cup
   - ☐ others: please, specify ☐

   (answer validation: only one box could be ticked.)

2. Where do you usually fill your client's medication organization devices? (please tick only one box)?
   - ☐ in the home care service's premises
   - ☐ in the care recipient's premises

(answer validation: only one box could be ticked.) (If „in the care recipient's premises“ was ticked, the survey continued with section VI)

V.

1. How do you usually monitor that medication organization devices have been filled correctly?
   - ☐ the nurse filling the medication organizer checks for conformity with the medication list herself/himself
   - ☐ a second nurse monitors conformity while / after filling by the first nurse
   - ☐ a second nurse checks for conformity in the care recipient's premises

   (answer validation: only one box could be ticked)

VI.

1. How many pharmacies serve as suppliers of medication that you fill into medication organization devices? *(answer validation: numbers > 0 were accepted)*

2. How many different medications do you fill into a medication organization device for an average care recipient? *(answer validation: numbers > 0 were accepted)*

3. How much time (in minutes) do you need to fill the medication of an average care recipient into medication organization devices that last for one week? Please include the time required for documentation and exclude the time needed to organize prescriptions, receive medication supply and deliver the filled medication organizers to clients, if applicable.
   (answer validation: numbers > 0 were accepted)

VII. This study is also intended to investigate the importance of various criteria for your home care nursing service when providing long-term medication to your clients and to research how well your current practices meet these criteria. Please answer the following questions:

1. How important are the following criteria for your homecare nursing service when providing long-term medication to your care recipients?
   - cost effectiveness
   - quality of care / error avoidance
   - flexibility in case of medication changes
   - limitation of liability risks
   - nurses‘ motivation
   - nurses‘ medication knowledge
   - straightforwardness of processes
   - compliance with hygiene standards

Answer options for each criterion entailed „not at all important”, “of little importance”, “neutral”, “important”, “very important” and “no opinion/don't know”. *(sequence of criteria was randomized)*

2. How well do your current practices of providing long-term medication to your clients meet the criteria?
    - cost effectiveness
    - quality of care / error avoidance
    - flexibility in case of medication changes
    - limitation of liability risks
    - nurses‘ motivation
    - nurses‘ medication knowledge
    - straightforwardness of processes
    - compliance with hygiene standards

Answer options for each criterion entailed „very poor", "poor", "fair", "good", "very good" and "no opinion/don't know". *(sequence of criteria was randomized)*

VIII. Having a pharmacy fill a disposable medication organization device poses an alternative to your current practice of filling devices yourself. This study investigates to what degree this alternative is known among homecare nursing services and what attitudes homecare nursing services exhibit towards disposable, pharmacy-filled medication organization devices. Please answer the following questions:

1. Are you familiar with the concept of disposable, pharmacy-filled medication organization devices?
    - ☐ No, I have not come across the concept before filling this survey
    - ☐ I have heard of the concept before, but I have no clear notion of what it means
    - ☐ Yes, I have a clear notion of what the concept means

    (answer validation: only one option could be ticked. If one of the first two options was ticked, the survey continued with section X, otherwise with section IX)

IX.

1. Which of the following options best describes your attitudes towards disposable, pharmacy-filled medication organization devices?
    - ☐ I feel skeptical about disposable, pharmacy-filled medication organization devices
    - ☐ I feel neither skeptical nor positive about disposable, pharmacy-filled medication organization devices
    - ☐ I feel positive about disposable, pharmacy-filled medication organization devices

    (answer validation: only one option could be ticked)

2. Could you substantiate your attitudes towards disposable, pharmacy-filled medication organization devices with a few key words? *(free text field)*

3. If you were to switch to disposable, pharmacy-filled medication organization devices, how would this in your opinion affect your invoicing of health insurances?
   - ☐ I do not know whether and what changes in our invoicing would result
   - ☐ We could still invoice the filling of medication organization devices, but we would potentially have to pay a fee to the pharmacy
   - ☐ We could no longer invoice filling of medication organization devices

   (answer validation: only one option could be ticked; sequence of answer options was randomized)

X. Finally, the study seeks to understand whether differences in the medication practices of home care nursing services are related to location, ownership or size of the entity. Please answer the following questions:

1. What is the population of the community where your homecare nursing service is located?

- ☐ >100.000
- ☐ 20.000 to less than 100.000
- ☐ 5.000 to less than 20.000
- ☐ < 5.000

(answer validation: only one option could be ticked)

2. In which state is your homecare nursing service located?
   - Pulldown menu with Baden-Wuerttemberg | Bavaria | Berlin | Brandenburg | Bremen | Hamburg | Hesse | Mecklenburg-Western Pomerania | Lower Saxony | North Rhine-Westphalia | Rhineland-Palatinate | Saarland | Saxony | Saxony-Anhalt | Schleswig-Holstein | Thuringia

(answer validation: only one option could be selected)

3. Into what category of ownership does your homecare nursing service fall?

- ☐ public
- ☐ non-profit
- ☐ private

4. To how many care recipients does your homecare nursing service cater?
   (answer validation: numbers > 0 were accepted)
